# Book Reviews

**Published:** 1894-06

**Authors:** 


### *BOOK REVIEWS.*

---

MEDICAL JURISPRUDENCE, FORENSIC MEDICINE AND TOXICOLOGY. By R. A. Witthaus, A.M., M.D., Professor of Chemistry and Physics in the University of the City of New York, and Tracy C. Becker, A.B., LL.B., Professor of Medical Jurisprudence in the University of Buffalo. Volume I. William Wood & Co., New York.

This is the first volume of a work which promises to become a valuable reference book on medical jurisprudence. It will deal exhaustively with all the subjects embraced under its title; more so than has ever yet been done in our country.

The volume begins with an introduction in which the history of forensic medicine and toxicology is thoroughly presented, from the most remote records to the present time.

The next chapter, by Professor Becker, is of interest to every physician. It discusses the "legal relations of physicians and surgeons, including their legal duties and obligations, their right to compensation, their privileges and duties as witnesses in court, and their liability for malpractice."

In the next chapter Mr. Boston, of New York, gives the law, with numerous references, concerning the confidential communications between physician and patient. The next chapter is a synopsis of laws in the United States and Canada, regulating the practice of medicine and surgery. It shows that only a few States remain without some protective legislation. Other chapters discuss fully the legal status of the dead body, the duties of coroners, the medico-legal consideration of wounds, medico-legal autopsies, determination of the time of death, death by heat and cold, medico-legal consideration of death by suffocation, medico-legal relations of electricity, personal identity, etc.

Three other volumes of this work will follow. The second and third volume will relate to questions concerning both dead bodies and living persons. The application of the microscope to medical jurisprudence will be treated of in the second volume. The fourth volume will contain the division relating to toxicology. (Introduction.)

A TEXT-BOOK OF THE DISEASES OF WOMEN. By Henry J. Garrigues, A.M., M.D., Professor of Obstetrics in the New York Post-Graduate Medical School and Hospital; Gynecologist to St. Mark's Hospital; Obstetric Surgeon to the New York Maternity Hospital, etc. With 310 engravings and colored plates. W. B. Saunders, 925 Walnut St., Philadelphia.

The *raison d'être* of this book has been to meet the needs of those physicians not having had the advantage of hospital training and who go off to post-graduate schools to learn gynecology. The author's aim has been to write a *practical work*, and his aim has been eminently successful. Much space is devoted to anatomy and physiology, more to the former than we think absolutely necessary. Theoretical discussions are omitted, and pathology has been treated very briefly. The idea of the author has been to enable the reader to make a diagnosis in a given case, and to teach him how to treat the disease. The book contains many ideas and suggestions that are new and not found in other works, many of these being of the most practical character. The part on "Treatment in General' is especially of this nature, describing many small, but important, points which are often passed over, on the supposition, probably, that everybody knows them anyhow. In the portion of the work devoted to operative gynecology the best recognized operations are carefully described, with the free use of illustrations. Diseases of the ovaries and tubes are described in some detail, and this will be found to be one of the best portions of the work.

We take pleasure in commending this book as one of the most practical, conservative and helpful that we have ever seen.

---

A MODERN WIZARD. By Rodreguez Ottolengui, author of "An Artist in Crime" and "A Conflict of Evidence." G. P. Putnam's Sons. New York. 1894.

This is a novel. The "hero" is a New York doctor. The story deals much with hypnotism, theosophy, metempsychosis and known and unknown medical science. In the trial of the doctor for poisoning his first wife the experts testified about the presence of morphine in the stomach and intestines, yet none was shown to have been taken by the mouth, but it was introduced hypodermically. No one in the court recognized the physiological error. The author, of course, is unaware of it. Aluminum hypodermic cases and medical tablets were not in use twenty years ago, and the hypodermic syringe has come into general use since.

The doctor owned the dome of an antediluvian temple, the alleged domicile of Æsculapius, of whom he claimed to be a lineal descendant. The walls were covered with hieroglyphics, showing medical knowledge, the acquirement of which placed the doctor twenty years in advance of his colleagues.

Finally our hypnotist and exponent of hieroglyphic knowledge reaches the point (killing his second wife with the diphtheria poison) where he thought it necessary to the good of others that he try a final experiment. He injects beneath his skin a substance derived from the brain of a person dying insane and becomes a maniac. He called the substance Sanatoxine. Despite the errors and the rather wild theories advanced it is a very interesting story.

M. B. H.

---

A TEXT-BOOK ON DISEASES OF THE EYE. By Henry D. Noyes, A.M., M.D., Professor of Ophthalmology in Bellevue Medical College, etc. Second and revised edition. 816 pages, illustrated by five chromo-lithographic plates, ten plates in black and colors, and 269 wood engravings. William Wood & Co., New York.

The second edition of Dr. Noyes's work is almost a new work in comparison with the first edition of 1881. Numerous additions have been made, and every part of the old edition has been brought fully up to the times in every respect. The part on cerebral anatomy and pathology, as pointed out by the author, is perhaps the most thorough of any book issued by an American author. A part of the old edition has been rewritten, and parts modified to conform with the most recent discoveries in the science of ophthalmology. The chapters on the anatomy and physiology of the optic nerve and brain connected with it are the most complete we have seen, and the tabulated pathological lesions from remote organs, and systemic disturbances, accidents, etc., is of great value to the reader who, in routine practice, is often liable to entirely overlook such elements as being disturbing factors in cases of diseases which one is too prone to attribute to local causes entirely.

The mechanical part of the volume is well executed. The illustrations are numerous and good. G. B.

---

ANTISEPTIC THERAPEUTICS.—By Dr. E. L. Trouessart, Paris, France. In two volumes. Physicians' Leisure Library Series. Price, fifty cents. George S. Davis, publisher, Detroit, Mich.

TREATMENT OF TYPHOID FEVER.—By D. D. Stewart, M.D., Philadelphia. Physicians' Leisure Library Series. Price, twenty-five cents. George S. Davis, publisher, Detroit, Mich.

The first volume of Antiseptic Therapeutics gives a very complete list of antiseptic agents used in medicine, with their chemical description and therapeutic uses. The second volume gives in more detail the antiseptic treatment of various diseases, such as those of the respiratory tract, the digestive apparatus, the genito-urinary tract, the eye, the skin, etc., and also the application of antiseptics to gynecology and midwifery.

In the Treatment of Typhoid Fever the author presents a very excellent description of the present status of our therapeutic measures in this disease, with some emphasis on the Brand method and the antiseptic line of treatment.

---

Mr. W. B. Saunders announces as in *active preparation* his NEW AID SERIES OF MANUALS FOR STUDENTS AND PRACTITIONERS.

The *Saunders' Aid Series* will not merely be condensations from present literature, but will be ably written by well-known authors and practitioners, most of them being teachers in representative American colleges. This *new series,* therefore, will form an admirable collection of advanced lectures, which will be invaluable aids to students in reading in comprehending the contents of "recommended" works.

Each manual, comprising about 250 pages, will further be distinguished by the beauty of the *new* type; by the quality of the paper and printing; by the copious use of illustrations; by the attractive binding in cloth; and by the extremely low price, which will uniformly be $1.25 per volume.

